# Optimizing Clubroot Management and the Role of Canola Cultivar Mixtures

**DOI:** 10.3390/pathogens13080640

**Published:** 2024-07-31

**Authors:** Andrea Botero-Ramirez, Brennon Kirk, Stephen E. Strelkov

**Affiliations:** 1 Department of Biological Sciences, Faculty of Arts and Science, MacEwan University, Edmonton, AB T5J 4S2, Canada; 2 Department of Agricultural, Food and Nutritional Science, University of Alberta, Edmonton, AB T6G 2P5, Canada; brennon@ualberta.ca

**Keywords:** clubroot, canola, disease management, cultivar mixtures, host resistance

## Abstract

The sustainable cultivation of canola is under threat from clubroot disease (*Plasmodiophora brassicae*). The pathogen’s resting spores can survive in the soil for extended periods, complicating disease management. Therefore, effective clubroot control requires a combination of tactics that provide multiple layers of protection. Management strategies have focused on pathogen avoidance and reducing disease levels in infested fields. The sanitation of machinery and field equipment remains the most effective method for preventing the pathogen’s introduction into non-infested fields. For disease reduction, crop rotation, liming, chemical control, and host resistance are commonly employed, with the use of clubroot-resistant cultivars being the most effective to date. However, resistance breakdown has been observed within four years of the introduction of new cultivars, jeopardizing the long-term effectiveness of this approach. A promising yet underexplored strategy is the use of cultivar mixtures. This approach leverages mechanisms such as the dilution effect, the barrier effect, induced resistance, disruptive selection, and the compensatory effect to control the disease. Cultivar mixtures have the potential to reduce the impact of clubroot on canola production while preserving pathogen population structure, thereby minimizing the likelihood of resistance breakdown. Given its potential, further research into cultivar mixtures as a management strategy for clubroot disease is warranted.

## 1. Introduction

Canola (oilseed rape; *Brassica napus* L.) is one of the most important oilseed crops worldwide, second only to soybean (*Glycine max* L. Merr.). Annual production is approximately 85 million metric tons globally [1]. Unfortunately, clubroot disease, caused by the soilborne protozoan *Plasmodiophora brassicae* Wor., represents a major threat to the sustainable cultivation of this crop. The occurrence of clubroot has been confirmed in over 80 countries, causing significant concerns due to its potential for rapid spread and economic losses [2]. The increasing importance of clubroot is reflected by a surge in scholarly attention, as evidenced by the rising number of publications on this topic. Clubroot-related publications have increased from just 41 in 1992 to 566 in 2022 [3].

Typical clubroot symptoms include the formation of large galls on the roots of infected susceptible hosts, which disrupt water and nutrient uptake, leading to plant wilting, stunting, and ultimately, to severe yield reductions or even plant death [4,5,6]. Clubroot is more severe in acidic soils when pH levels are between 5.0 and 6.0 [7,8,9,10], soil moisture is high [11,12,13,14], and temperatures are between 20 °C and 25 °C [15,16,17,18,19,20,21]. Yield losses attributable to this disease increase proportionally with increases in the disease severity index (DSI) and can reach as high as 100%. It is estimated that each 1% increase in DSI can result in a 0.49% to 1% yield loss under ideal conditions for pathogen development [22]. Clubroot management can be challenging, primarily due to the longevity of the pathogen’s resting spores, which have a half-life of approximately 4 years [23] but can persist in the soil for up to 17 years [24]. Consequently, various practices have been explored for control of this disease, including crop rotation, biological control, liming of the soil, application of soil fumigants and fungicides, and manipulation of seeding dates [25]. However, the most effective approach for disease management remains the utilization of clubroot-resistant cultivars [26]. Nevertheless, no single practice offers a sustainable solution for clubroot control, particularly in broad-acre crops like canola. This is demonstrated by the rapid emergence of *P. brassicae* populations capable of overcoming the resistance in canola cultivars in Canada, first identified only four years after the introduction of the resistance trait [27].

Ideally, the management of clubroot and other plant diseases should rely on a combination of tactics that can reinforce each other and provide multiple levels of protection if one should fail. Additionally, novel management strategies should be considered and explored. One approach, which has shown promise in other pathosystems [28,29,30,31,32] but has received limited attention in clubroot, is the deployment of cultivar mixtures. Such mixtures may have the potential to reduce disease pressure and favor the longevity of resistance. The aim of this review is to provide an overview of clubroot and its management, with a focus on cultivar mixtures and their potential advantages and limitations in controlling this disease.

## 2. Life Cycle of *P. brassicae*

The life cycle of the clubroot pathogen comprises three main stages: (i) survival in the soil as resting spores, (ii) primary infection, and (iii) secondary infection. Primary infection commences with the germination of resting spores, releasing primary biflagellate zoospores. This germination is stimulated by the soil microbial community, which can be modulated by factors such as root exudates of host and non-host plants [33,34,35,36]. Following germination, the zoospores swim through water films towards root hairs and root epidermal cells [37]. Upon encountering the cell wall of a root hair or epidermal cell, the zoospores penetrate the cells and form uninucleate primary plasmodia within one day of infection [37,38,39].

The primary plasmodium then undergoes division to produce a zoosporangium, which, in turn, produces new (secondary) zoospores. These secondary zoospores can be released into either the rhizosphere, subsequently infecting the host cortical tissue (a process taking about 7 days), or move to neighboring cortical cells to initiate the secondary infection. This marks the onset of the third stage of the pathogen life cycle, which begins with the formation of intracellular secondary plasmodia [39].

The nuclei in the secondary plasmodia undergo cruciform division, and the plasmodia enlarge to occupy the intracellular space of the colonized cortical cells [37]. It has been proposed that upon maturation, further meiotic divisions, and cytoplasmic cleavage occur to produce haploid multinucleated resting sporangial plasmodia [37]. At this stage, unorganized growth and disintegration of the vascular and root tissue occur, leading to the formation of galls, the typical clubroot symptoms [40,41]. Subsequent divisions of the secondary plasmodia and the deposition of cell wall material result in the formation of new resting spores, which are released into the soil as survival structures when the galls decompose [42].

## 3. Management Strategies

Clubroot management in canola is based mainly on the use of clubroot-resistant cultivars. However, resistance breakdown has been observed within four years of the introduction of new cultivars, jeopardizing the long-term effectiveness of this approach [27,43,44]. Research suggests that diversifying and introducing new resistance sources through the development of cultivars with polygenic resistance and gene pyramiding, along with the use of multilines and cultivar mixtures, can delay the resistance breakdown caused by strong selection pressure [45,46,47]. In canola, efforts are ongoing to enhance the durability of clubroot-resistant canola by incorporating various sources of monogenic and polygenic resistance through gene pyramiding to reduce disease levels and increase resistance durability. However, the extended survival of *P. brassicae* resting spores in the soil and the potential for virulence shifts in pathogen populations present significant challenges for clubroot management. Consequently, various other disease control strategies have been explored [25], typically aiming to achieve two main objectives: (i) preventing the introduction of the pathogen into previously non-infested fields, and (ii) minimizing the incidence and severity of clubroot disease in crops cultivated in infested fields.

### 3.1. Pathogen Avoidance

Considering the soilborne nature of *P. brassicae*, the most effective management strategy is exclusion through the sanitization of farm machinery and other equipment [25,48,49,50]. Equipment sanitation involves removing soil, crop debris, and other organic residue from equipment and vehicles, as well as applying chemical disinfectants to remove any residual inoculum [51]. While sanitation is an effective method for curtailing the spread of *P. brassicae*, its implementation presents challenges. Inactivating the pathogen’s resting spores is difficult, as chemical disinfectants developed for managing bacteria, viruses, and fungi may not effectively target protist resting spores. While numerous chemicals have been tested for their efficacy in inactivating *P. brassicae* resting spores, only a few have proven to be effective. Bleach (sodium hypochlorite), Spray Nine (n-Alkyl [5% C12, 60% C14, 30% c16, and 5% C18]), n-Alkyl (32% C14 and 68% C12), and dimethyl benzyl ammonium chloride (0.15%) have demonstrated the highest effectiveness, with efficacies ranging from 95% to 99% [51]. However, farmers may face constraints in adopting sanitation practices due to the significant time and effort required to clean machinery in an adequate manner [52].

### 3.2. Clubroot Management in Infested Fields

After a field becomes infested with *P. brassicae*, reducing the incidence and severity of clubroot can involve implementing multiple practices, including: (i) crop rotation [52,53,54,55,56], (ii) soil liming [57,58,59], (iii) application of fungicides or soil fumigants [25,53], and (iv) deployment of resistant cultivars [60,61,62]. Most of these practices were developed for use in vegetable brassica crops, and generally, except for genetic resistance, they tend to be too expensive or impractical for broad-acre crops such as canola [63,64].

#### 3.2.1. Crop Rotation

While the benefits of crop rotation in breaking pathogen life cycles and reducing inoculum buildup are widely acknowledged [65,66], this strategy proves less effective against soilborne pathogens such as *P. brassicae*, which produces long-lived survival structures [66]. Studies indicate that despite the longevity of resting spores, their population levels in the soil can decline by up to 90% after a 2-year break from canola, subsequently stabilizing [56,67] and resulting in a Type III survivorship curve [68]. As such, a minimum 2-year interval with non-host plant species between canola crops, combined with the use of resistant cultivars, has been recommended to mitigate the impact of clubroot on canola [53,67]. However, in highly infested soils, more than 2 years might be necessary to achieve commercially acceptable yields in susceptible canola cultivars [53,54,69,70]. In addition to its impact on pathogen inoculum density, the adoption of crop rotation strategies is recommended to mitigate resistance breakdown. Successive cropping of clubroot-resistant cultivars has been associated with pathotype shifts towards more virulent pathogen strains, emphasizing the need for longer rotations [71,72].

#### 3.2.2. Liming

Soil pH significantly influences clubroot development, with the disease typically being more severe in acidic soils [7,8,48]. Various studies have found that clubroot is favoured at pH values between 5.0 and 6.0, reduced at pH ≥ 7.0, and eliminated at pH > 8.0 [7,8,9,10]. Nevertheless, severe clubroot symptoms can sometimes occur in alkaline soils, particularly under high resting spore loads and favourable moisture and temperature [20,73,74,75]. Therefore, modifying the soil environment to create less favorable conditions for clubroot often involves increasing the soil pH through lime application [26,57]. Different types of lime include dolomitic limestone (CaCO_3_•MgCO_3_), calcitic limestone (CaCO_3_), calcium oxide (CaO), and hydrated lime (Ca(OH)_2_) [76]. While increasing the soil pH may provide some protection against clubroot, it is unlikely to be sufficient as a standalone measure for disease prevention, especially under high inoculum pressure and favorable environmental conditions for disease development [14,20,73].

To maximize the benefits of liming, it is recommended to maintain a soil pH above 7.2, with more favorable outcomes observed at pH values as high as 8.0 [7,8,10]. However, achieving such alkaline pH levels often requires large quantities of lime, which may be prohibitively expensive and impractical to source, apply, and/or incorporate over multiple fields annually [77]. Another limitation of liming is the inconsistency in clubroot control obtained [57,58,62,78]. For example, hydrated lime can reduce disease severity up to 91% in susceptible canola some years, but may be ineffective in disease-conducive environments [57].

The efficacy of liming is influenced by several factors that affect its capacity to achieve a uniform and timely increase of soil pH. These factors include: (i) the extent of mixing of lime with the soil, (ii) the fineness of the lime particles, (iii) the residual basicity or acidity of nitrogen sources in the rhizosphere, (iv) the time of the year when it is applied, and (v) the timing of application in relation to the crop [62,78,79,80].

#### 3.2.3. Chemical Control

The application of fungicides can be an attractive strategy for clubroot management [53]. Fungicides such as fluazinam, cyazofamid, cyazofamid + methiadinil, and flusulfamide applied before seeding or as seed treatments can effectively reduce disease levels and increase yields in various crops including broccoli (*Brassica oleracea* var. *italica*), cauliflower (*Brassica oleracea* var. *botrytis*), cabbage (*Brassica oleracea* var. *capitata*), Chinese cabbage (*Brassica rapa* var. *pekinensis*), and canola. These fungicides may be incorporated into the soil through banding, broadcasting, spot drenching, or root irrigation [81,82,83,84,85,86]. In canola, quintozene incorporated into the soil has also been reported to reduce clubroot severity [83].

Seed treatments with the fungicides azoxystrobin, thiamethoxam + difenoconazole + metalaxyl + fludioxonil, flusulfamide, clothianidin + carbathiin + trifloxystrobin + metalaxyl, and carbathiin + thiram have been evaluated for controlling clubroot on canola [87]. Although these treatments showed promise under greenhouse conditions, they were not broadly effective under field conditions in western Canada. This lack of effectiveness is likely due to their protective effects not persisting long enough to prevent infection from the soil inoculum [87]. While some fungicides have demonstrated efficacy against clubroot, it is currently improbable that any fungicide applied to soil would receive approval for use, especially on the scale required for canola cultivation [62].

Alongside fungicides, there has been interest in using soil fumigants for clubroot management. Soil fumigants contain active ingredients with a relatively high volatility and a low water solubility, and demonstrate activity against a wide range of soil microflora and microfauna [88]. However, fumigants such as chloropicrin (trichloronitromethane), dazomet, metam sodium, methyl bromide, and methyl isothiocyanate have shown inconsistent results for controlling clubroot on canola [25]. In contrast, they appear to be more effective at reducing the disease in vegetable crops such as cabbage, cauliflower, Chinese cabbage, Shanghai pak choi (*Brassica rapa* var. *chinensis*), and swede (*Brassica napus* var. *napifera*) [88,89,90,91].

In canola, soil fumigants applied at high dosages, including metam sodium (0.4 to 0.8 t of active ingredient (a.i.) ha^−1^), vapam (78 to 622 L ha^−1^), and dazomet (0.4 and 1.6 L ha^−1^), have demonstrated effectiveness in reducing clubroot severity under both greenhouse and field conditions [92,93,94,95]. However, soil fumigants can be highly toxic and costly to apply. Therefore, their widespread adoption is unlikely for canola production systems, except for use in targeted management of isolated infection foci [93,94,96].

#### 3.2.4. Host Resistance

Genetic mapping of clubroot resistance (CR) genes has been extensively conducted in *Brassica* species to address resistance to *P. brassicae.* In *B. napus,* only a few major CR genes and some quantitative trait loci (QTL) have been identified [97,98,99,100]. Significant efforts have been made to map resistance sources in allied species, including *B. rapa* and *B. oleracea*, to enable CR introgression into *B. napus* [101,102].

In *B. rapa*, at least 10 major resistance genes controlling clubroot response have been identified and mapped [103,104,105,106,107,108,109,110,111,112,113,114]. These genes often confer pathotype- or race-specific resistance to *P. brassicae* [101]. In contrast, resistance in *B. oleracea* is mainly quantitative, and controlled by a few major genes along with multiple quantitative trait loci (QTLs) with both major and minor effects [115,116,117,118,119,120,121]. The CR genes, mostly from the A-genome of *B. rapa*, have been used for breeding clubroot-resistant canola [111,122,123]. However, the number of genes introgressed into *B. napus* is limited, primarily due to difficulties in interspecific hybridization, including hybridization barriers, hybrid sterility, and challenges in recovering a stable euploid lines with the resistance gene [102]. This limitation has affected the availability of CR genes in commercial canola cultivars.

Despite these challenges, the deployment of clubroot-resistant cultivars remains one of the most effective disease management practices [25]. The majority of canola cultivars available in Canada appear to carry a single resistance gene derived from the European winter oilseed rape ‘Mendel’ [124]. This cultivar originated from a resynthesized *B. napus* line obtained from the cross of the *B. oleracea* line ‘ECD 15’ and the *B. rapa* line ‘ECD04’, which was then crossed with the winter canola cv. ‘Falcon’ [125]. ‘Mendel’ carries a major dominant gene and two recessive genes against clubroot, and was proven to confer resistance against the most prevalent pathotypes of *P. brassicae* in Europe and Canada up to 2011 [111,126,127]. Given its availability and its capacity to confer resistance against multiple pathotypes, ‘Mendel’ served as the basis for the development of the first group of clubroot-resistant cultivars in the Canadian Prairies [126].

Cultivars carrying resistance derived from ‘Mendel’ possess what is commonly known as ‘first-generation’ resistance [128]. Given the emergence of new *P. brassicae* pathotypes able to overcome first-generation resistance [27,129], new canola hybrids with novel resistance traits have been developed. Cultivars with new clubroot-resistance traits possess what is termed ‘second-generation’ resistance [43], the genetic basis of which is proprietary and may differ across hybrids. Unfortunately, evidence shows that clubroot resistance in some second-generation canola cultivars has been overcome in certain fields in western Canada [44].

In Canada, the first CR canola cultivar (‘45H29’) was released in 2009 [26], and currently, 73 out of 95 registered canola cultivars are designated as clubroot-resistant [130]. While planting of resistant cultivars remains the most efficient and convenient method for clubroot management, the extensive cropping of CR canola applies significant selection pressure on the pathogen [131,132].

*P. brassicae* populations are very diverse, with multiple pathotypes often present in a single field and even within a single gall [132,133]. Consequently, the continuous cultivation of CR cultivars can lead to an increase in the abundance of rare, resistance-breaking pathotypes within the population. This selection pressure results in shifts in the virulence of the *P. brassicae* populations, favoring the emergence of pathotypes capable of breaking or overcoming resistance, as observed in Canada and elsewhere [27,43,50,129,134,135].

To mitigate resistance breakdown, diversification of the gene pool in cultivated canola has been suggested as a crucial strategy. Two approaches can be used for that purpose: gene pyramiding and the use of cultivar mixtures. Gene pyramiding involves stacking multiple resistance genes in a single cultivar, an approach that is believed to enhance the durability of CR genes when compared to deploying single genes sequentially [136]. On the other hand, in cultivar mixtures, multiple cultivars with different resistance genes are grown together in a single field [32]. Modelling studies suggest that both strategies are equally effective at reducing pathogen inoculum density and spread, and in maintaining resistance over time [137]. Despite the conceptual appeal of gene pyramiding, its immediate application is challenging, as it may require the introduction of multiple CR genes from allied species into *B. napus.* If the introgression of a single gene is difficult, pyramiding multiple genes is even more complex due to interspecific hybridization limitations, potential differential expression of genes in different backgrounds, linkage drag, and the challenge of combining multiple resistance genes into a single plant genotype quickly. Additionally, epistatic interactions may result in one resistance gene masking the effects of others [47,136,138]. While gene pyramiding has gained wider acceptance as a strategy to improve the durability of clubroot resistance, the use of cultivar mixtures should not be dismissed and warrants further exploration.

## 4. Cultivar Mixtures as an Option for Disease Management

The deployment of resistant cultivars may not always be a feasible option for clubroot management, as it presents several disadvantages [139]. Firstly, the limited availability of currently effective CR genes poses a challenge, restricting the possibilities for incorporating multiple genes into individual lines. Secondly, the widespread monocropping of clubroot-resistant cultivars carrying single CR genes can lead to virulence shifts in pathogen populations, resulting in a decrease in their effectiveness, as has already been observed with clubroot of canola [27,43] and other pathosystems [139]. This underscores the crucial role of diversity in modern agroecosystems for disease management.

Introducing diversity, either temporally or spatially, is critical for improving disease management. Temporal diversification, accomplished through practices such as crop rotation, rotating resistance genes, or implementing cover cropping, serves as one approach. Alternatively, spatial diversification can be achieved by integrating cultivar mixtures, intercropping techniques, or enhancing species diversity across fields, via techniques including the formation of mosaic patterns [140,141].

Cultivar mixtures, which promote increased pathogen diversity, increase the likelihood of avirulent pathotypes occurring on a given host genotype. This phenomenon is likely to enhance induced resistance, while simultaneously reducing the selection pressure towards more virulent, resistance-breaking pathotypes [29]. Cultivar mixtures can take various forms, including multiline and isoline mixtures featuring genetically identical cultivars differing only in singular resistance genes or other specific characteristics, as well as cultivar mixtures [142]. Cultivar combinations occupy the midpoint between intercropping and monocropping, enhancing diversity while mitigating the complexity associated with managing intercrops [143]. The objectives behind these mixtures guide cultivar selection, with an emphasis on aspects such as weed control, insect control, soil fertility, yield, abiotic stress tolerance, or disease control [144]. Designing objective-based mixtures provides flexibility in addressing combinations of these goals based on specific needs. An increased emphasis on crop diversity through cultivar mixtures extends benefits to growers beyond disease management. Diverse agricultural communities exhibit a higher productivity and temporal stability compared with equivalent yet low-diversity systems [143,145].

### 4.1. Mechanisms of Control

Studies have identified five control mechanisms for managing crop diseases through the implementation of cultivar mixtures: dilution effects, barrier effects, induced resistance, disruptive selection, and compensatory effects [146]. The extent to which each mechanism comes into play depends on the specific crop and disease. However, the main objective remains mitigating pest pressure to reach and maintain an acceptable threshold.

**Figure 1:**
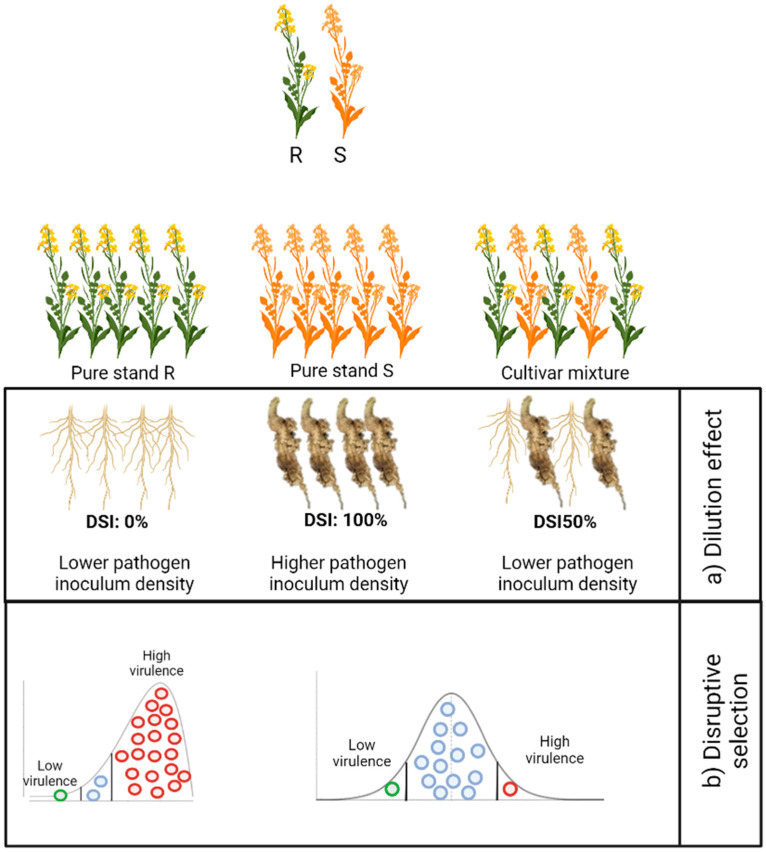
Illustration of the mechanisms of control for soilborne diseases using cultivar mixtures: (**a**) dilution effect and (**b**) disruptive selection. (**a**) The dilution effect occurs when a lower density of susceptible plants reduces the probability of infection compared to pure stands. (**b**) Disruptive selection maintains a diverse array of pathotypes due to a varied population of hosts, balancing low-virulence pathotypes with highly virulent ones, rather than selecting exclusively for resistance-breaking pathotypes. DSI, disease severity index. (Created with Biorender.com [148]).

Dilution effects occur when there is a lower density of susceptible plants in the field, leading to a reduced probability of infection (Figure 1). In the context of mixtures, only a fraction of the inoculum spreads among the various components, resulting in decreased disease severity and lower inoculum levels [139,142]. Understanding the dispersal mechanisms of a pathogen is crucial for estimating the effectiveness of dilution effects in limiting disease spread. Dilution effects from utilizing cultivar mixtures are maximized when inoculum is spread equally among different host genotypes. For example, pathogens disseminated in the soil, such as *P. brassicae*, will undergo fewer dispersal events per growing season compared to wind- or splash-dispersed pathogens like *Zymoseptoria tritici* (Septoria leaf blotch of wheat) or *Leptosphaeria maculans* (blackleg of canola) [147].

Mixing susceptible and resistant cultivars creates pathogen refuges, potentially reducing the emergence rate of resistance-breaking pathotypes [149]. However, while this may exert selection pressure towards numerous pathotypes of low to intermediate virulence, it does not eliminate resistance-breaking events. Disruptive selection limits the epidemic development of specific pathogen strains with an affinity for specific cultivars due to the spatial distribution of cultivars, thereby reducing the selection towards specific pathotypes [150]. Therefore, promoting a diverse pathogen population, encompassing multiple pathotypes rather than populations with singular or a few highly virulent pathotypes, has the potential to prolong the lifespan of existing resistance genes. These advantages also reduce the likelihood of pathogens developing fungicide resistance [150]. Nevertheless, refuges within cultivar mixtures may prove ineffective against pathogens with long-lived resting spores, such as *P. brassicae*. Susceptible individuals persist as detrimental inoculum bridges, sustaining disease pressure and maintaining field inoculum loads [149].

**Figure 2:**
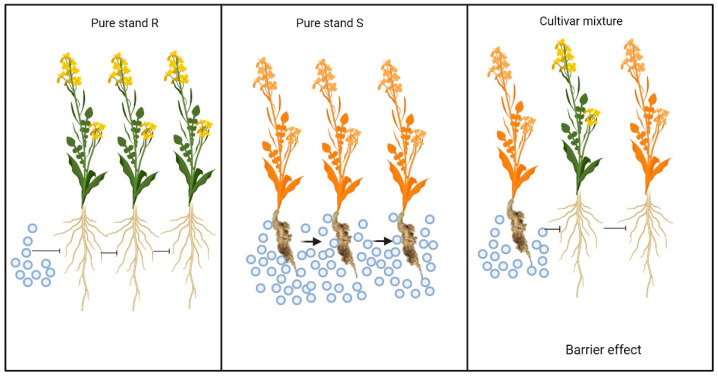
Illustration of the barrier effect as a mechanism of control for soilborne diseases. This effect involves interrupting pathogen spread through the presence of non-host plants. When the pathogen inoculum encounters a resistant plant, its spread is halted, preventing it from finding suitable hosts and infection sites. (Created with BioRender.com).

Barrier effects refer to the interruption of pathogen spread through the presence of non-host plants, like those with alternative resistance genes or of a different crop species in intercropping systems (Figure 2). When the pathogen inoculum encounters a resistant plant, its spread is halted, preventing it from finding suitable hosts and infection sites [147]. Barrier and dilution effects are more evident when crop rotations and mixtures are applied at the landscape level, creating mosaics that enhance biodiversity on larger scales. This dispersion of susceptible individuals in both space and time enhances the effectiveness of these effects [142,146]. For example, phenotypic variation within mixtures, such as differences in leaf angle and plant height, can induce barrier effects by generating uneven canopies that interrupt foliar pathogens’ dispersal and germination [150].

Mixtures of susceptible, moderately resistant, and resistant genotypes maintain the genetic diversity of pathogen populations by promoting the coexistence of virulent and avirulent pathotypes. In cultivar mixtures, induced resistance occurs by the activation of the plant immune response to avirulent pathogen inoculum, preventing or limiting further infections by virulent spores. The protection is based on the activation of defense mechanisms and metabolic changes that accelerate pathogen recognition [140,146,150].

Disruptive selection occurs in mixed stands, leading to shifts in pathogen populations towards a diverse array of pathotypes. This tends to maintain a balance of low virulence pathotypes alongside highly virulent ones, rather than a shift towards only resistance-breaking pathotypes (Figure 1) [146]. Disruptive selection drives shifts away from specialized, highly virulent pathotypes towards more generalist, less virulent ones that may be more easily managed [139,140,142,146]. In fields with multiple host genotypes and numerous pathotypes, the theoretical occurrence of inter-pathotype competition for available susceptible host tissue could contribute to a reduction in disease severity within cultivar mixtures [151]. By modifying the selection pressure on pathotypes, cultivar mixtures exploit existing trade-offs between fitness and reproductive rates, maintaining high virulence pathotypes at lower frequencies compared to monocrops [152].

However, selection pressure and subsequent evolutionary drift will vary based on the pathogen’s reproduction rate and type (sexual or asexual) [140]. Reproductive rate becomes an important consideration in cultivar mixtures, as the more rapidly a host’s maximum disease load is reached, the less effective mixtures become [142]. Additionally, incorporating multiple resistance genes within mixtures reduces the likelihood of a pathogen mutation capable of overcoming all of them [153].

Compensatory effects describe how individual diseased plants reduce competition with individuals able to overcome disease, therefore stabilizing or improving yields. These effects differ between species and are related to overall phenotypic plasticity [150]. They become more pronounced with increasing complementarity among mixture components, where niche partitioning and variable interactions between each species and the environment result in greater productivity of the entire plant stand [143,144,154]. Realistically, inter-cultivar mixing compatibility and intraspecific variation must be considered to produce effective mixtures.

Combining cultivars into mixtures offers numerous opportunities to mitigate epidemics and enhance the durability of resistance, by offering multiple mechanisms of disease mitigation, such as dilution effects, barrier effects, disruptive selection, compensatory effects, and induced resistance. Complimentary cultivars, each with different resistance genes and phenotypic traits, serve as alternative points of function against resistance breaking. Leveraging the strengths of all combined cultivars alongside management practices like resistance-gene rotation can prove to be an effective strategy for reducing input costs and disease levels.

### 4.2. Designing Cultivar Mixtures

Selecting cultivars to include in mixtures involves numerous considerations due to the absence of clear, standardized selection criteria. Barot et al. (2017) [144] outlined two main approaches for cultivar mixture assembly: trait-based and trait-blind. Trait-based mixtures entail selecting cultivars based on specific qualities, such as disease mitigation, yield improvement, lodging control, or drought tolerance, to design groups of cultivars with specialized functions. In contrast, trait-blind methodologies select candidates from available high-performing cultivars without specific traits in mind, estimating their mixing compatibility beforehand. In trait-blind selection, cultivar mixtures must outcompete their monocrop counterparts [142]. While trait-based approaches are more appealing, they require extensive phenotyping databases and knowledge of the resistance, which may be proprietary or unavailable. Mixing ability is complicated further by considerations such as the capacity to distinguish between cultivars in multi-cultivar fields, where managing fewer cultivars is more feasible [31].

Cultivar mixtures should strive for agronomic complementarity, focusing on factors such as the presence of resistance genes, leaf and canopy architecture, seed mass, plant height, maturity class, anthesis period, root architecture, mycorrhizal dependence, and drought tolerance, among others [143,144,147]. To optimize the benefits of mixtures, it is essential to select genotypes with consistent phenotypic traits, such as plant height, leaf angles, branching patterns, and maturity schedules, to minimize intraspecific competition [142,143,147,155]. By reducing intraspecific competition and maximizing niche partitioning through cultivar selection, the environmental impact can be minimized and resource utilization optimized, benefiting the entire stand [144]. However, the complexity of designing mixtures is influenced by phenotype plasticity, which is influenced by the environment and neighboring plants [144,156]. Therefore, plasticity can both enhance and diminish cultivar complementarity.

Disease severity typically decreases as the number of components in a mixture increases, assuming they possess varying resistance traits [151]. Previous research has examined the influence of susceptible–resistant plant ratios (S:R ratios) on disease control, particularly in barley powdery mildew (*Blumeria graminis* f. sp. *hordei*), wheat stripe rust (*Puccinia striiformis* f. sp. *tritici*), and septoria tritici blotch (*Z. tritici*). Maintaining a 1:3 ratio of susceptible and resistant plants has been recommended for effective protection against foliar diseases [30,157,158]. When it comes to cultivar mixtures, epidemiologists suggest the optimal number varies depending on the resistance genes in the plant and the pathogen’s lifecycle. Recent studies highlight that the types of resistance (functional diversity) are more important than the number of cultivars (genetic diversity) in disease control [146]. In reality, it may be challenging to find a perfect cultivar mixture that can resist all pathotypes across multiple diseases. Therefore, it may be more pertinent to compensate for the susceptibilities of one cultivar with the resistance gene(s) of others [143]. The selection of ‘key traits’ for mixing also varies depending on the objective of the mixture. For example, mixtures designed to combat clubroot could consider factors such as resistance genes and root architecture, and might differ from mixtures aimed at controlling a windborne disease like leaf blotch, where canopy characteristics are more relevant.

In addition to considering trait complementarity and the presence of resistance genes, selecting the optimal number of cultivars in mixtures is crucial for effectively managing diseases while maintaining or even improving overall yield [146,159]. However, determining the appropriate number of cultivars in a mixture depends on the specific crop species and the targeted disease. According to Mikaberidze et al. [151], the ideal number of components is the one that reduces disease severity to an economically acceptable level. This optimal number is influenced by the degree of pathogen specialization, indicating that fewer components should be included when dealing with highly specialized pathogens, while a greater number of genotypes may be necessary for less specialized ones. Therefore, considering the number of cultivars is a nuanced and context-dependent aspect of plant disease management.

Furthermore, it is important to emphasize that the diversity of resistance genes is more important than genetic diversity when determining the number of cultivars in a mixture [160]. It is essential to align the pathotypes present carefully with the resistance genes utilized in a mixture. This task becomes increasingly challenging when dealing with quantitative resistance in cultivars, as the combination of both qualitative and quantitative resistance can have complex and potentially interactive effects [146]. Central to these considerations are the definitions of “resistant” and “susceptible”, which are often subjects of debate and vary based on the crop, pathogen, and previous data, particularly in pathosystems lacking gene-for-gene interactions [31].

### 4.3. Benefits

Cultivar mixtures offer numerous advantages for crop production, including reduced input costs, improved disease management, potential for higher yields, yield stability, and additional ecological benefits. The objectives of cultivar mixtures, such as pest control, yield optimization, soil fertility enhancement, and disease management, can be customized based on regional conditions and the availability of cultivars, providing flexibility to meet agronomic requirements [144,149]. By slowing disease progression and reducing disease pressure, cultivar mixtures can decrease the need for fungicide applications, thereby saving growers money and minimizing selection pressure on pathogen populations for developing resistance [150]. This slower disease progression can also extend the fungicide application window, enabling growers to take advantage of optimal weather conditions.

Building on this approach, the incorporation of cultivar mixtures alongside crop rotations further limits the likelihood of disease outbreaks. By interspersing strongly resistant host cultivars with moderately resistant or susceptible counterparts, this strategy demonstrates its potential to extend the efficacy of cultivars, even in cases where resistance has been previously overcome [142]. This approach holds promise for sustainable disease management within the framework of cultivar mixtures, contributing to the resilience and longevity of agroecosystems. In a study by Montazeaud et al. [161], 202 random dual cultivar mixtures of durum wheat inbred lines were selected from a pool of 179 candidates resistant to septoria leaf blotch (*Z. tritici*), resulting in a 4% increase in yield and a 17% reduction in disease severity compared to pure stands. In instances of adverse abiotic stress, cultivar mixtures can stabilize yields through niche partitioning, matching or surpassing the performance of monocrop systems [144,154].

Cultivar mixtures offer secondary benefits in the form of ecological services such as weed suppression, improved soil fertility, and support for pollinators and natural enemies of pests [143,154]. The unintended advantages of mixtures, such as weed suppression, contribute to improved grower returns and yields by reducing interspecific competition [154,162]. By suppressing weeds, including pathogen-susceptible volunteers or closely related species that serve as inoculum bridges, cultivar mixtures can further maximize benefits. The plasticity in the deployment and design of mixtures also offers growers numerous advantages depending on their agronomic needs. While these benefits are evident in small-scale experiments, mixtures are believed to be more efficient at larger scales more familiar to growers [142]. In contrast to the lengthy process of traditional resistance breeding, cultivar mixtures can be updated readily as new cultivars emerge, ensuring that mixtures remain current and build upon existing management practices.

### 4.4. Challenges

While cultivar mixtures offer many advantages, they also present drawbacks, primarily due to antagonistic effects resulting from unsuitable pairings among a wide range of candidate cultivars. Pairing cultivars with contrasting characteristics, such as varying plant heights, has been shown to increase competition between cultivars, with taller varieties outcompeting shorter ones and ultimately reducing the overall yield [147,163]. However, recent studies suggest that this might not always be the case [164]. Additionally, it is important to recognize that under field conditions, a single crop species can face pressure from multiple pathogens, a factor that is frequently overlooked. Modeling efforts have struggled to account for the complexity of dealing with multiple diseases, and field trials have shown that the interplay of various diseases can significantly affect the yield stability of cultivar mixtures [146,158].

While over-yielding is often observed in cultivar mixtures, the outcomes can vary, with yield increases typically ranging from 2% to 5%. Occasionally, antagonistic effects may occur, leading to yield reductions and increased disease severity [159,161].

Past trials have been conducted under specific cropping conditions, including high disease pressure and low fungicide inputs [146]. The disparity between experimental scenarios and “real-world” field conditions presents significant challenges for integrating cultivar mixtures into industry practices. Further research is needed to establish the generalizability of previous findings to diverse pathosystems.

A major concern in utilizing cultivar mixtures to delay resistance breakdown is the potential selection of “super-pathogens” that can overcome all the resistance genes included in the mix [29]. Under exponential pathogen reproduction, virulent mutations that can defeat all resistance genes within the cultivar mixture may arise, albeit with a low probability [150]. Given the high rate of resting spore production by *P. brassicae*, it is important to consider carefully the cultivars to include in the mixture, to minimize the likelihood of selection for pathotypes capable of overcoming current resistance genes.

Further research is necessary to identify the traits that make cultivars compatible beyond simple parameters like expected yield, crop height, and maturity ratings, particularly under variable field conditions such as soil type, disease prevalence, and climate. Resistance gene pyramiding and stacking within cultivar mixtures have proven effective in combating disease progression [139,143,152]. However, introgressing multiple resistance genes within and between compatible mixture cultivars presents significant challenges, including being time-consuming and requiring extensive screening [144]. The potential to capitalize on stacking available resistance genes with alternative genetic modes of action in creating cultivar mixtures is hindered by proprietary information among seed companies.

Continuous updates are essential for mixture combinations derived from high-performing candidates or those possessing specific disease resistance. The advantages of mixtures are optimized when deployed against specific diseases and pathotypes in fields with known histories. However, growers may struggle to identify appropriate mixtures because pathotypes at the field scale are typically unknown, leading to generalized approaches based on regional data from disease surveys, which may not fully realize the benefits of mixtures.

Environmental characteristics such as temperature and drought tolerance, shifts in pathogen and pest populations, breeding advancements, and market demands also require cultivar mixtures to be flexible enough to adapt. Currently, no simple methods or universal guidelines are available to formulate mixtures in response to these evolving conditions [143]. Additionally, it is unclear how to predict which mixtures will have synergistic, additive, antagonistic, or non-effect outcomes, underscoring the necessity of mixture guidelines. It is important to note that while mixtures may enhance the durability of resistance, reduce disease severity, or counteract the emergence of “super-pathogens”, aligning these benefits with established agronomic practices may not always be feasible [152].

A current obstacle to cultivar mixture adoption is its effective marketing. Precision agriculture has introduced new technologies, helping growers to enhance crop management and maximize agricultural outputs. However, these technologies are designed primarily on monocrop imagery, indicating that adaptations are necessary for their application to cultivar mixtures [144]. Additionally, growers may choose to select candidate cultivars and pre-mix them before seeding, allowing them to tailor mixtures according to specific agronomic requirements. Nevertheless, confusion persists regarding mixtures and seeding practices, particularly in optimizing the spatial arrangement of cultivars to maximize benefits such as niche partitioning and combatting disease progression [146].

Moreover, growers may choose not to plant cultivar mixtures if all mixture components already exhibit a range of disease resistances. In such cases, they might opt for a single high-performing cultivar that is regionally adapted, has a clear resistance rating, a predictable yield, and ensures a uniform harvest [149]. Regional regulatory obstacles may also hinder the acceptance of non-uniform products [143]. Overall, the implementation of cultivar mixtures faces various challenges. Nevertheless, they present a viable strategy for an integrated pest-management approach.

## 5. Potential for Clubroot Management

Clubroot presents a significant challenge to canola production in western Canada. Despite the limited use of cultivar mixtures for controlling soilborne diseases in the past [157], there is an opportunity to leverage this practice to mitigate the spread and impact of clubroot. By employing cultivar mixtures with multiple CR genes, growers can potentially decrease disease levels, reduce pathogen populations, and alleviate selection pressure on these populations, offering an additional avenue for clubroot management [165].

To maximize the durability of CR genes in cultivars, strategies should aim to limit the selection of more virulent pathogen strains and reduce their population sizes [165]. For instance, studies in wheat have demonstrated that a mixture of susceptible and resistant cultivars in a 1:3 ratio can decrease the incidence of the soilborne wheat mosaic virus (SBWMV) transmitted by the plasmodiophorid *Polymyxa graminis* by approximately 40% compared with a pure stand of the susceptible cultivar [166]. Similarly, in soybean, research has shown that 1:1 mixtures of resistant and susceptible cultivars can reduce cyst densities in soil infested with soybean cyst nematodes. Additionally, these mixtures help to prevent shifts in the nematode population towards greater virulence, thus extending the effectiveness of resistant cultivars [28].

While the primary approach to clubroot management involves using resistant cultivars, relying solely on this approach raises sustainability concerns. Continuous planting of resistant cultivars exerts significant selection pressure on pathogen populations, ultimately leading to resistance breakdown [165]. Resistance breakdown occurs when new, more virulent pathotypes emerge through mutations or when existing pathotypes with virulence alleles capable of overcoming the CR genes of the cultivated varieties are selected [165,167]. During infection cycles, mutation events can generate new pathogen variants with a competitive advantage over the strains initiating the infection. Intra-host competition may result in the development of highly virulent pathogens, leading to a shift in the mean virulence of the population [167]. However, these events are rare. Drake et al. [168] calculated that the rates of spontaneous mutations in microorganisms, including plant pathogens, are typically close to 1/300 per genome per replication.

Given that *P. brassicae* may produce up to 1 × 10^10^ resting spores per gram of galled root [23], the emergence of new pathotypes through the mutation of virulence genes is plausible. However, genetic analyses of ‘novel’ resistance-breaking pathotypes of *P. brassicae* in Canada revealed that these pathotypes likely already existed in the pathogen population at very low frequencies, rapidly increasing in response to selection pressure from repeated exposure to host resistance [131,132]. These findings suggest that resistance breaking in *P. brassicae* is more likely a result of intensive selection pressure from the widespread use of resistant cultivars, leading to an increase in the frequency of resistance-breaking pathotypes, rather than being attributable solely to mutations.

Designing cultivar mixtures for clubroot management in canola requires a thorough understanding of pathogen biology and access to a diverse genetic pool of the host to ensure targeted and effective control. The clubroot-resistant canola cultivars available in the Canadian market are classified into first-generation cultivars, which typically possess a single CR gene derived from ‘Mendel’, and second-generation cultivars, which contain undisclosed resistance traits that may vary across hybrids. This lack of detailed knowledge regarding the genetic basis for clubroot resistance in available cultivars complicates the generation of trait-based cultivar mixtures. Therefore, an initial, partially trait-based approach may be necessary until comprehensive labeling for clubroot resistance is implemented across canola cultivars.

The selection of cultivars to incorporate into mixtures should initially include a combination of resistant and susceptible cultivars. Ideally, mixtures should contain susceptible cultivars, first-generation clubroot-resistant cultivars, and second-generation clubroot-resistant cultivars. However, there are currently very few susceptible canola hybrids available on the market, making it technically challenging to incorporate them into the mix. Since the resistance in first- and second-generation cultivars appears to be different, mixing these two types of cultivars could effectively extend the lifespan of available resistance sources.

Fortunately, a list of currently available cultivars in Canada, along with their classification into first- and second-generation resistance categories, is available [169]. Additional phenotypic information needed to ensure agronomic compatibility is also accessible from small plot and field scale data collected from Canola Performance Trials [170]. It is also essential to consider traits beyond clubroot resistance, such as herbicide tolerance (important for conservation tillage), height, days to maturity, lodging, shattering rates, and oil content, among others. These traits are crucial for effective crop management practices, including pesticide applications for diseases like sclerotinia stem rot (*Sclerotinia sclerotiorum*) and harvest management.

Further research is required to determine the optimal susceptible–resistant cultivar ratios and the ideal number of cultivars in a mixture to achieve effective disease control while maintaining crop profitability. Nonetheless, a 1:3 ratio of susceptible and resistant cultivars is worth exploring, given its success in various pathosystems, including the soilborne wheat mosaic virus (SBWMV) transmitted by *P. graminis* [166], and foliar diseases such as barley powdery mildew, wheat stripe rust, and septoria tritici blotch [30,157,158]. This mixture could serve as an effective starting point for determining cultivar mixtures aimed at managing clubroot, ideally incorporating both first- and second-generation resistance types. Including at least one susceptible cultivar alongside first- and second-generation clubroot-resistant cultivars could promote greater diversity in common *P. brassicae* pathotypes and encourage inter-pathotype competition. Field *P. brassicae* populations are known to consist of multiple pathotypes [171], and resistance breakdown is often attributed to the rapid increase of individuals from resistance-breaking pathotypes already present in the population at low frequencies [131]. Therefore, by maintaining a population of susceptible plants mixed with plants harboring multiple CR genes, it is likely that selection pressure will be reduced, thereby preserving the overall population structure.

Given the prevalence of *P. brassicae* pathotypes 3A, 3H, and 3D in the Canadian prairies [43], designing mixtures should focus on selecting cultivars with resistance genes effective against these pathotypes. Incorporating cultivars with resistance to multiple dominant pathotypes or rotating mixtures with specific resistance genes can enhance the impact of disruptive selection and increase the durability of CR genes. Given the proprietary nature of second-generation resistance sources, incorporating multiple resistance genes to combat common pathotypes will further improve the effectiveness of cultivar mixtures due to a broader foundation of genetic resistance. However, additional research is needed to determine if the virulence of *P. brassicae* can shift due to disruptive selection, which may lead to a transition from high virulence and resistance-breaking pathotypes to more manageable generalist ones [139,140]. Research may also help elucidate the effect(s) of common pathotypes on canola cultivar mixtures, by evaluating potential yield or over-yielding benefits. Moreover, a multiple pathogen approach could be considered when generating mixtures, by including canola cultivars with varying levels of resistance not only to clubroot, but also to sclerotinia stem rot, blackleg, and other important diseases of this crop.

## 6. Conclusions

Clubroot causes significant yield losses in canola and represents a major threat to the sustainable production of this crop. The use of cultivar mixtures may represent an effective approach for mitigating the effects of this disease. Historically, cultivar mixtures have been primarily explored in cereals to manage wind-borne diseases, achieve over-yielding under adverse conditions, and understand the dynamics of competition between mixture components. Applying these principles to the clubroot pathosystem in canola will require establishing optimal susceptible–resistant cultivar ratios, maximizing niche partitioning, and enhancing resistance-gene complementarity. Additionally, identifying the most effective mechanisms for disease reduction in these mixtures will also be important for the sustainable application of this approach. Ultimately, the strategic use of cultivar mixtures in canola may provide a viable method for enhancing clubroot control, prolonging the effectiveness of resistance genes, and managing *P. brassicae* populations. Further research will be needed to refine these strategies to realize their potential in clubroot management.

## Data Availability

Data are contained within the article.
